# Phylogenetic Analysis of the vesicular fusion SNARE machinery revealing its functional divergence across Eukaryotes

**DOI:** 10.6026/97320630014361

**Published:** 2018-07-31

**Authors:** Gagandeep K. Khurana, Poonam Vishwakarma, Niti Puri, Andrew Michael Lynn

**Affiliations:** 1 School of Life Sciences, Jawaharlal Nehru University, New Delhi, India- 110067; 2 School of Computational and Integrative Sciences, Jawaharlal Nehru University, New Delhi, India-110067

**Keywords:** SNAREs, clade, Phylogeny, transmembrane domain, paralogs

## Abstract

Proteins of the SNARE (Soluble N-ethylmaleimide-sensitive factor attachment protein receptors) family play a significant role in all vesicular fusion events involved in endocytic and exocytic pathways. These proteins act as molecular machines that assemble into tight four-helix bundle complex, bridging the opposing membranes into close proximity forming membrane fusion. Almost all SNARE proteins share a 53 amino acid coiled-coil domain, which is mostly linked to the transmembrane domain at the C-terminal end. Despite significant variations between SNARE sequences across species, the SNARE mediated membrane fusion is evolutionary conserved in all eukaryotes. It is of interest to compare the functional divergence of SNARE proteins across various eukaryotic groups during evolution. Here, we report an exhaustive phylogeny of the SNARE proteins retrieved from SNARE database including plants, animals, fungi and protists. The Initial phylogeny segregated SNARE protein sequences into five well-supported clades Qa, Qb, Qc, Qbc and R reflective of their positions in the four-helix SNARE complex. Further to improve resolution the Qa, Qb, Qc and R family specific trees were reconstructed, each of these were further segregated into organelle specific clades at first and later diverged into lineage specific subgroups. This revealed that most of the SNARE orthologs are conserved at subcellular locations or at trafficking pathways across various species during eukaryotic evolution. The paralogous expansion in SNARE repertoire was observed at metazoans (animals) and plants independently during eukaryotic evolution. However, results also show that the multi-cellular and saprophytic fungi have limited SNAREs.

## Background

The evolutionary transition from prokaryotes to multicellular eukaryotes had led to the internal compartmentalization of the cell and emergence of the endomembrane system. The transport of proteins, lipids and other macromolecules between these compartments occurs via packaging of vesicles with cargo from the donor organelle membrane and its fusion with the target membrane. These cargo-laden vesicles have integral membrane proteins termed as v-SNAREs, which form, complex with the cognate t-SNAREs on the target membrane. SNAREs are a family of small cytoplasmically oriented evolutionarily conserved proteins involved in protein trafficking pathways i.e., trafficking of newly synthesized proteins, turnover of preexisting proteins and organelle morphogenesis. Functionally these proteins were classified into vesicle-associated v-SNAREs and target membrane associated t-SNAREs. All SNARE proteins are comprised of 53-63 amino acid residue long SNARE domain, an extended coil coiled segment arranged in heptad repeats, which is directly adjacent to a transmembrane domain at the C-terminal end. The SNARE motifs from complementary sets of SNARE proteins zipper up into a tight 4-helix bundle complex between opposing membranes in trans-configuration [1, 2]. This brings the membranes into close proximity, overcomes the repulsive forces and provides the driving force to initiate membrane fusion. In the interior of this four-helix bundle, 16 virtual layers are formed from highly conserved hydrophobic residues, which are intervened by an ionic layer in the center, consisting of three glutamine (Q) residues and one arginine (R) residue. This leads to the classification of SNARE proteins into Q and R SNAREs. These SNAREs can be classified in to Qa, Qb, Qc, and R families with the exception of Qbc family members that contribute two SNARE motifs to the SNARE complex. Further these were grouped into twenty subgroups based on subcellular localization in the respective organelles and regulation of different trafficking pathways [3].

**Figure 1:**
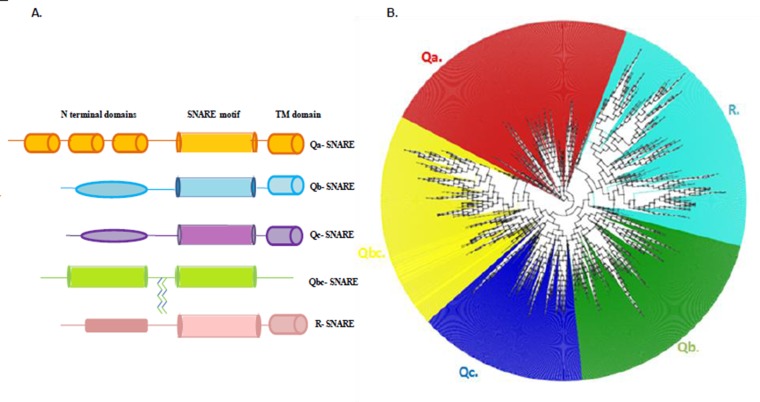
Structural classification and Phylogenetic reconstruction of the SNARE super family using Fast tree 2. (A). Schematic representation of the structural classification of SNARE protein family based on domain architecture. All SNARE proteins have an α- helical coiled-coil domain (cylinder) that is involved in the formation of a parallel four-helix bundle, which brings the membranes into close apposition and triggers their fusion. Syntaxins (orange) Qb SNARE (blue), Qc SNARE (purple) and vesicle-associated membrane proteins (VAMPs, pink) each contributing one helix, while SNAP25-like proteins (green) contribute two helices to the SNARE complex. (B) The outline of the unrooted Phylogenetic tree reconstructed from 2197 protein sequences for the entire SNARE super family. The SNARE protein sequences were retrieved using perl script from the SNARE database http://bioinformatics.mpi bpc.mpg.de/snare/index.jsp and aligned using MAFFT and MUSCLE software. The result of the Multiple Alignment was used to construct Maximum Likelihood (Neighbor joining method) using Fast tree 2. The reconstructed tree resolved into four well supported clades reflecting the position of SNARE domains in the four helix complex - Qa (red), Qb (green), Qc (blue) and R (light blue) with the Qbc (yellow) constituting the fifth clade that contributed two domains to the SNARE complex.

Qa SNARE family includes almost all known Syntaxins while a few Syntaxins are categorized as Qc SNAREs. Almost all Syntaxins consist of N-terminal regulatory Habc domain linked to the H3 SNARE domain and end in the transmembrane domain. Most of the Qb SNAREs (except Sec20) and Qc SNAREs have three helix forming N-terminal domains linked to a single SNARE domain and finally a transmembrane domain at the Cterminal end [4]. Likewise R-SNAREs consist of an N-terminal variable domain followed by the SNARE domain that finally ends in a transmembrane domain (Figure 1A). R-SNAREs can be classified into short Vesicle associated membrane proteins (VAMPs) or brevins and long VAMPs or longins depending on whether they possess a short and variable domain or a conserved longin domain of 120-140 amino acids at their N-terminus respectively [5]. The longin R-SNARE subgroup consists of Sec22, Ykt6, and VAMP-7 that share an N-terminal extension termed as longin domain having a profilin like fold structure consisting of β-sheets with parallel and anti-parallel α- helices on either side [5]. The R-SNAREs can be further grouped into RD and RG SNAREs. The RD SNAREs consist of centrally conserved Arginine (R) and adjacent Aspartic acid (D) residues in the SNARE domain and include mainly neuronal brevins while RG SNAREs consist of centrally conserved Arginine (R) and Glycine residue (G) in the SNARE domain [5].

In addition to evolutionarily conserved SNARE domain, these proteins harbor specific signatures i.e., K/HDEL retrieval in the ER localized SNAREs [6], NPF motif in the Golgi localized Sed5 [7], YGRL in TGN localized Syntaxin 6 [8], dileucine (LL) motif in Endosome localized Syntaxin 8 [9], CAAX motif in Golgi localized R-SNARE Ykt6 [10], FXFXD motifs in the TGN localized Qb SNARE Vti1 [11] etc.

Early findings had reported an elaborate SNARE repertoire in Last eukaryotic common ancestor (LECA), which remains conserved throughout the eukaryotic evolution [12, 3, 13]. Interestingly the SNARE repertoire evolved through the duplication and diversification of basic SNARE proteins followed by lineage specific sculpting across various eukaryotic subgroups. Previous phylogenetic studies had been performed for each of the 20 SNARE subgroups of the SNARE superfamily in metazoans revealing a significant increase in endosomal and Plasma membrane SNAREs during evolution [14]. An independent increase has been reported in the secretory and endocytic SNAREs in plants through detailed phylogenetic analysis [15]. This increase might be linked to the transition from unicellular to multicellular eukaryotic lifestyle. By contrast in depth phylogenetic survey in fungi suggested that multicellularity in fungi was achieved without an overall expansion in the SNARE repertoire [16]. Likewise many protists such as Paramecium harbor a large set of SNARE like proteins i.e., Synaptobrevin 8-12, SNAP-25 like as compared to flowering plants and metazoans [17]. SNARE proteins are ubiquitous in multicellular organisms, and well defined in the LECA. Therefore, it is of interest to understand the functional divergence of the SNARE family through the evolution of multicellularity. It is also of interest to estimate the correlation of multicellularity with the expansion in the SNARE repertoire in different eukaryotic lineages?

## Methodology

### Retrieval of sequences from SNARE database

For this study, 2197 SNARE protein sequences were retrieved from more than 200 species of metazoans (animals), plants, fungi and protists listed on the SNARE database http://bioinformatics.mpibpc.mpg.de/ snare/index.jsp after PERL script written in-house. The dataset include sequences for all five families and subgroups i.e., Qa-504, Qb -424, Qc-570, Qbc -285 and R SNARE subfamily-514.

### Sequence Alignment and Phylogenetic reconstruction

The sequences for each subgroup within the SNARE family were aligned using MAFFT software [18] and a master alignment (profile) was generated by merging the profiles for Qa ,Qb ,Qc, R and Qbc families through profile-profile alignment using MUSCLE software [19]. The alignments were viewed and edited using Jalview software [20]. The phylogenetic trees were reconstructed from edited alignment in Fasttree 2 software, which is freely available at http://www.microbesonline.org/fasttree [21]. This software utilizes CAT approximation as a model of rate heterogeneity across various sites (alignment columns) [21]. The proportion of invariable sites was estimated from data and the Jones, Taylor, and Thornton (JTT) distance matrix was used as a substitution matrix. The stopping rule of the algorithm was used, but the algorithm had to run for at least the suggested number of iterations. All other settings of the application were set to default values. Afterwards Maximum likelihood Mapping was used to calculate the local support value (confidence value) based on the Shimodaira-Hasegawa test for each edge of phylogenetic tree [21].

## Results & Discussion

Previous phylogenetic trees of the SNARE family have been separately constructed for each of the 20 SNARE subgroups independently [14]. The Phylogenetic tree reconstructed from the edited alignment performed for this work, segregated the entire SNAREs into five well separated clades i.e., Qa, Qb, Qc, R and Qbc families reflecting their position in the four helix SNARE complex (Figure 1B). In order to improve resolution, Qa, Qb, Qc and R family specific trees were also generated. Besides independent analysis of each family, the Qbc family was used as a reference with both the Qb and Qc families as it contains both the Qb and Qc domains.

### Phylogeny of Qa SNAREs (Syntaxins)

**Figure 2:**
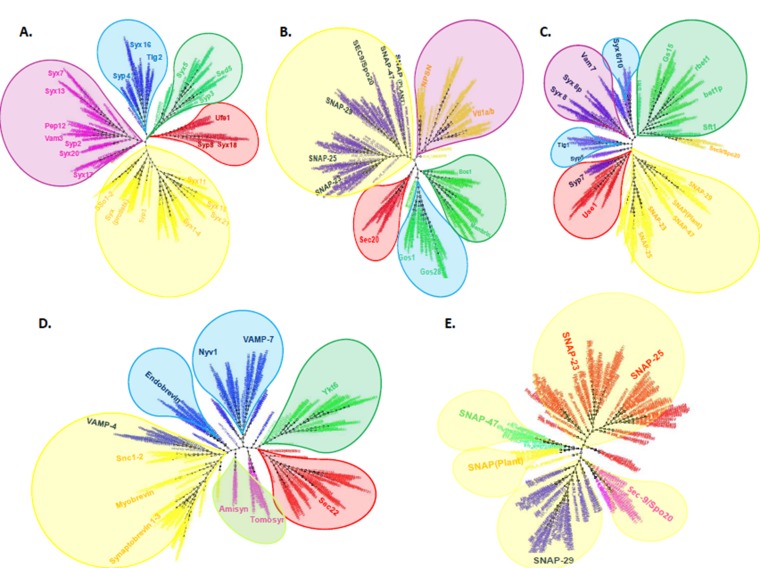
The outline of the unrooted Phylogenetic trees of Qa, Qb/Qbc , Qc/Qbc ,Qbc and R SNAREs reconstructed from SNARE protein sequences using Fasttree 2. (A). The Qa SNARE family segregated into five well supported organelle specific clades- ER localized Syntaxin 18 and its fungi ortholog Ufe1; Golgi localized Syntaxin 5 and its orthologs Sed5 (fungi) and Syp3 (plants); Trans Golgi localized Syntaxin 16 and its orthologs Tlg1 (fungi) and Syp4 (plants); Endosome localized Syntaxin 7, Syntaxin 13 and -20 and their orthologs Pep12 and Vam3 (fungi) and Syp2 (plants) and Plasma membrane localized Syntaxin 1-4, and their orthologs SSo1/2(fungi), Syp1(plants) and Syntaxin PM (protists). (B) The Qb SNARE family also segregated into five well supported organelle specific clades-ER localized Sec20; Golgi localized Membrin and its fungi orthologs Bos1; Trans Golgi localized Gos28 and its fungi ortholog Gos1; Endosome localized Vti1 and NPSN (plants and protists) and Plasma membrane localized SNAP-25 and its orthologs. (C) The Qc SNARE family segregated into five clusters based on their localization in respective organelles- ER localized Use1 and Syp7; Golgi localized Bet1, Gs15 and its fungi orthologs Sft1; Trans Golgi localized Syntaxin 6, Syntaxin 10 and its orthologs Tlg1 (fungi) and Syp5 (plants); Endosome (Vacuole) localized Syntaxin 8 and Vam7 (fungi) and Syp6 (plants)) and Plasma membrane localized SNAP- 25 (SNAP25c) and its orthologs. (D) The R SNARE family resolved into five clusters- ER localized Sec22; Golgi localized Ykt6; Endosome (Vacuole) localized VAMP7, Nyv1 (fungi specific); vesicular membrane localized Synaptobrevin 1-3, Myobrevin and Syc1-2, VAMP-4 and Regulatory R SNAREs Tomosyn and Amisyn (pink). The Trans Golgi Network (TGN) localized VAMP4 (blue) showed homology and clustered along with vesicle localized secretory brevins while the Endobrevin/ VAMP8 (blue) constituted a separate cluster (E) The Qbc SNARE tree resolved into metazoan specific SNAP-25, SNAP-23, SNAP-29 and SNAP-47; fungi specific paralogs Sec9/Spo20 and plant specific Qbc SNARE SNAP-33 (Plants). The metazoan specific SNAP-25 clustered along with SNAP-23 while SNAP-29 and SNAP-47 diverged off separately suggesting the origin of SNAP-23 from SNAP-25. The ER localized SNAREs are depicted with red bubble; Golgi localized with green bubble; Trans Golgi Network (TGN) with blue, Endosomes (vacuole) with pink; Plasma/vesicle membrane SNAREs with yellow bubble and Regulatory SNAREs with light green color bubble.

The Phylogenetic tree reconstructed from Qa SNAREs (Syntaxins) resolved into five organelle specific clades i.e., ER localized Syntaxin 18; Golgi localized Syntaxin 5; trans-Golgi Network localized Syntaxin 16; Endosome (vacuole) localized Syntaxin 7 and Plasma membrane localized Syntaxins 1-4 and their orthologs (Figure 2A).

The Endoplasmic Reticulum localized Qa SNARE segregated into metazoan specific Syntaxin 18 and fungi specific Ufe1 subgroups. The Golgi localized Qa SNARE clade segregated into metazoan specific Syntaxin 5, fungi specific Sed5 and plant specific Syp3. The Trans Golgi Network localized Qa SNARE clade further segregated into metazoan specific Syntaxin 16, fungi specific Tlg2 and plant specific Syp4. The Endosome (vacuole) localized Qa SNARE clade segregated into metazoan specific Syntaxin 7, Syntaxin 13, Syntaxin 20 and fungi specific Pep12 (prevacuolar compartment) and Vam3 (vacuole) while the plant specific Syp21 (prevacuolar compartment) and Syp22 (vacuole) diverged off separately (Figure 2A). Interestingly, we note that Syntaxin 17 (having two transmembrane domains and a hairpin structure) clustered together with endosomal Syntaxins, although this protein was found to be localized to the ER. Recent studies have shown that though this protein is part of the LECA repertoire, it has been lost in a number of lineages including yeast, and is now attracting widespread interest due to its role in autophagy, with a role beyond the initially assigned ER localization [22, 23, 24].

The plasma membrane localized Qa SNARE clade split into metazoan specific Syntaxin 1, 2, 3, 4, Syntaxin 11, 19 and 21; yeast specific Sso1 and -2; plant specific Syp1 and protist specific Syntaxins. Syntaxin 1 is involved in faster kinetics of exocytosis in neurons [25] whereas Syntaxin 2, 3 and 4 are associated with slower kinetics of release in non-neuronal cells. The vertebrate specific isoforms i.e., Syntaxin 11 and its paralogs Syntaxin 19 and -21 diverged and branched off separately [26] (Figure 2A).

### Expansion of Endosomal and Plasma membrane Syntaxins

Consistent with existing understanding, our results predict that the first round of whole genome duplication led to the expansion of endosomal (vacuole) SNAREs and some secretory SNAREs in higher eukaryotes while the second genome duplication event results in increase in Plasma membrane associated SNAREs. In metazoans, the endosomal SNARE Syntaxin 7 duplicated into Syntaxin 13 and later into Syntaxin 20 [14]. Likewise the yeast (fungi) Pep12 and Vam3p arose as a result of gene duplication within Saccharomycotina clade in fungi [16] while in plants Syp21 and Syp22 emerged as a result of an independent duplication event [15].

The Plasma membrane Syntaxin 1 diverged and evolved into set of secretory Syntaxins in metazoans i.e., Syntaxin 2, 3, 4 and further highly deviated paralogs i.e., Syntaxin 11 [26], Syntaxin 19 [27] and Syntaxin 21 in the lineage of vertebrates. However expansion in Syntaxin repertoire took place in plants independently and has been mainly associated with Plasma membrane Syntaxins Syp11-14 [15].

### Phylogeny of Qb SNAREs

The Phylogenetic tree reconstructed from Qb SNAREs resolved into five clades i.e., ER localized Sec20, Golgi localized Membrin and Bos1, trans Golgi Network localized Gos28 and Gos1, Endosome (Vacuole) localized Vti1 and NPSN. Qbc SNAREs constitute the plasma membrane localized members of this subfamily and are added for reference (Figure 2B).

The ER localized Sec20 clade split into metazoan, fungi and plant specific subgroups. This SNARE protein showed low homology with other SNAREs and is involved in retrograde transport from Golgi to ER [6]. Likewise, the cis-Golgi localized Qb-SNARE clade resolved into metazoan specific Membrin, fungi specific Bos1 and plant specific Membrin 11 and Membrin 12 while the trans Golgi Network localized Qb SNARE segregated into metazoan specific Gos28, fungi specific Gos1 and plant specific Gos11 and Gos12. The Endosome (vacuole) localized Qb SNAREs segregated into metazoan specific Vti1a/b, fungi specific Vti1p and the plant specific Vti11-14 and NPSN (Novel Plant SNARE). (Figure 2B).

### Evolution of Endosomal Qb SNAREs

In metazoans, the endosomal Vti1a and Vti1b are encoded by two separate genes; the former localizes to Golgi and TGN while latter has a role in early and late endosomal trafficking [28]. By contrast fungi Vti1p is encoded by a single gene Vti1 required for cell survival [16] whereas plants have multiple paralogs Vti11-to- 14 that perform multiple roles in the Golgi/Endosomal/Vacuole SNARE complexes [15]. Further another SNARE protein, NPSN (Novel plant SNARE) was initially discovered in plants, but now reported in basal fungi and protists. This suggests that NPSN present in LECA is retained in plants and lower eukaryotes i.e., protists and basal fungi but later has been completely lost from metazoans and higher fungi during evolution [29, 30].

### Phylogeny of Qc SNAREs

The Phylogenetic tree reconstructed from Qc SNAREs resolved into five clusters i.e., ER localized Use1 and Syp7; Golgi localized Bet1, Sft1 and Gs15; Trans Golgi Network localized Syntaxin 6 and Syntaxin 10, Endosome localized Syntaxin 8. As mentioned earlier in the Qb subfamily, plasma membrane localization is through the specialized Qbc subfamily containing both Qb and Qc domains, and is inserted into our analysis as a reference (Figure 2C). We note that in our results, the ER localized Use1 has been clustered together with Syp7 which has not been reported earlier. Use1 is specific to animals and fungi while Syp7 is reported in plants and protists [12]. The plant specific Syp7 has dual localization in the ER and Plasma membrane in Arabidopsis [31]. The Golgi localized Qc SNAREs segregated into Bet1, Sft1 and Gs15. The metazoan specific rbet1 clustered along with Gs15 while fungi specific bet1p clustered with Sft1. The TGN localized metazoan specific Syntaxin 6 and Syntaxin 10 constituted separate cluster while fungi specific Tlg1 and plant specific Syp5 diverged off separately. Likewise the Endosomal localized Syntaxin 8 diverged into metazoan and fungi specific Syntaxin 8 while fungi specific Vam7 clustered adjacent to the TGN localized paralogs in fungi i.e., Syntaxin 10p and Syntaxin 6p. By contrast, vacuole localized plant specific Syp6 formed a separate cluster. (Figure 2C).

### Evolution of Qc SNAREs

The TGN localized Qc SNAREs represent kingdom specific isoforms i.e., metazoan specific Syntaxin 6 and Syntaxin 10 SNAREs that arose after gene duplication in metazoans [14], Tlg1 is fungi specific that Syp5 has been reported in protists and plants [32]. The endosome localized Qc SNAREs Syntaxin 8 diverged into metazoan and fungi specific isoforms whereas plant specific Syp6 is endosomal and branched off separately, indicating an independent line of evolution [33]. Another vacuole localized SNARE Vam7 has been reported in fungi, which clustered adjacent to the Syntaxin 6 and Syntaxin 10. Although it is now reported that Syntaxin 10/6 homologs have also been found in fungi [34].

### Phylogeny of Qbc SNAREs

Qbc SNAREs are the only family of SNAREs consisting of dual SNARE motifs connected by a linker region. The Phylogenetic tree reconstructed from Qbc SNAREs segregated into SNAP-25, SNAP-23, SNAP-29, SNAP-47, Sec9 and SNAP (Plant). SNAP-23 showed homology and clustered along with SNAP-25 while SNAP-29 and SNAP-47 constituted separate clades. Likewise the fungi paralogs Sec9 and Spo20 clustered together while plant specific SNAP-33type SNAREs branched off separately (Figure 2E). SNAP-25 is a component of presynaptic complex of mammals and associated with faster kinetics in neurons while its ubiquitously expressed homolog SNAP-23 was reported only in vertebrates associated with slower kinetics in non-neuronal cells such as immune cells [35]. The two other Qbc SNAREs in metazoans include SNAP-29 involved in trafficking within Golgi apparatus and involved in constitutive secretion while SNAP-47 showed a widespread distribution on intracellular organelles [36]. In the Phylogenetic analysis, the clustering of SNAP-23 and SNAP-25 suggested that gene duplication of SNAP-25 in metazoans led to the emergence of SNAP-23 in the lineage of vertebrates [14]. Likewise the two paralogs in fungi i.e., Sec9p and Spo20p arose after gene duplication event within Saccharomycotina (yeast) lineage in fungi [16]. Although the two fungal paralogs clustered separately from metazoan equivalents of SNAP-25 yet it is believed that both of these evolved from a common ophisthokont ancestor [22]. Further the plant specific SNAP33-type proteins (SNAP Plant) including SNAP-33, SNAP- 28, and SNAP-34 clustered together and constituted a separate subclade [15] (Figure 2E).

### Phylogeny of R-SNAREs

The Phylogenetic tree reconstructed from R-SNAREs segregated into four well supported clades based on their localization in respective organelles i.e., Endoplasmic reticulum localized Sec22; Golgi localized Ykt6; Endosome localized VAMP-7 and Nyv1; vesicle localized Synaptobrevin 1-3, Myobrevin and Snc1/2.The VAMP-4 clustered together with other vesicle associated RG brevins while Endobrevin constituted a separate cluster. However, the regulatory R-SNAREs Tomosyn and Amisyn segregated into two separate branches (Figure 2D). The Endoplasmic reticulum localized R SNARE Sec22 clade segregated into metazoan, fungi and plant specific subgroups. Likewise the golgi localized Ykt6 segregated into metazoan, fungi and plant specific subgroups. The endosome localized SNAREs split into metazoan specific VAMP-7, fungi Nyv1 and plant specific VAMP-71 and VAMP-72. Interestingly, the Endobrevin (RG-brevin) proteins constituted a separate cluster, which has not been seen in previous analysis. These proteins are implicated in secretion of zymogen granules, and are not simply endosome localised. The vesicle-localized brevins bifurcated into neuronal RD brevins and non-neuronal RG brevins. The neuronal brevins including Synaptobrevin 1-3, SybA/B and n-Syb in Drosophila constituted a separate cluster while non-neuronal RG brevins i.e., Myobrevin, Snc1/2 and VAMP-4 clustered separately. The regulatory R-SNARE Tomsyn and Amisyn constituted two separate branches within the phylogenetic tree. These SNAREs completely lack transmembrane anchor hence cannot serve as fusogenic R-SNAREs but instead regulated accessibility of SNARE acceptor complex for the R-SNAREs residing on secretory vesicles (Figure 2D).

### Evolution of R SNAREs

Longins i.e., Sec22, Ykt6 and VAMP-7 had been reported in LECA eukaryotic ancestor and are conserved across all the eukaryotes [5]. The Endosomal SNAREs consist of VAMP-7 and fungi specific Nyv1. In the phylogenetic analysis, Endobrevin constituted a separate cluster adjacent to Endosomal SNAREs. However previous phylogeny suggested the emergence of Endobrevin from VAMP-7like SNARE in lower metazoans after loss of N-terminal profilin like fold. Likewise the endosomal SNAREs in green plants consist of VAMP-71 and VAMP-72. The green plants completely lack 'brevins' [5]. In order to compensate the green plants developed another plant specific VAMP-72 in addition to the canonical VAMP-71 [15]. The evolutionary younger 'brevins' split into RD and RG brevins. Though 'VAMP- 4' is implicated in trafficking towards TGN, it clustered with other RG-brevins as compared with previous phylogeny, which suggested they might have their common origin.

## Conclusion

A comprehensive phylogenetic analysis confirms that the basic SNARE repertoire including twenty SNARE subgroups was conserved in LECA. The expansion of SNARE repertoire in metazoans and plants confers an evolutionary advantage by allowing for provision of more versatile and orchestrated endosomal and secretory trafficking pathways. However, this expansion cannot be correlated with the rise of multi-cellularity as most of the SNAREs remained as single copy in fungi. Endoplasmic reticulum and Golgi localized SNAREs are singletons in most eukaryotes and their secretory pathways are preserved [14]. Nonetheless, persistent SNARE duplications were also found in the endosomal and secretory pathways [14]. Many of these duplications in the Plasma membrane SNAREs lead to functional paralogs associated with differential kinetics of exocytosis especially in metazoans. These include the emergence of SNAP-23 (Slower kinetics of exocytosis) from SNAP-25 (faster kinetics); Syntaxin 2, 3, 4 from Syntaxin 1 (faster calcium triggered exocytosis) and divergence of longins and brevins (faster kinetics of exocytosis) during eukaryotic evolution. The global phylogenetic tree reconstructed from profile-profile alignment of SNARE superfamily effectively resolved SNAREs into four well-supported clades representing Qa, Qb, Qc, Qbc and R families. This also mirrors the similar conservation observed among the vast majority of Rab GTPases; where functional equivalence has been retained although function and location are not in agreement [37]. Each of the Qa, Qb, Qc and R-SNARE family specific tree effectively separated into organelle specific clades such as ER, Golgi, Trans Golgi, Endosomes and Plasma membrane. These were later split into lineage specific clades. TGN localized R-SNARE VAMP-4 clustered with secretory brevins, granules localized VAMP-8 constituted a separate cluster, and Syntaxin 17 although reported as a second ER Syntaxin [23] clustered with endosomal Qa SNAREs with evolving specialized functions. Thus, SNARE proteins revealed evolutionary homology and most of the orthologues are useful as intracellular markers in secretory and endocytic pathways.

## Conflict of interest

The authors have no conflict of interest.

## Abbreviations

SNARE: Soluble N-ethylmaleimide-sensitive factor attachment protein receptors
VAMP: Vesicle associated membrane proteins
ER: Endoplasmic reticulum
TGN: Trans Golgi Network
PM: Plasma membrane
Stx/Syx: Syntaxin
Syb: Synaptobrevin
Syp: Syntaxin of plants
MSA: Multiple Sequence Alignment
Gs27: Golgi SNARE with a size of 27 kilodaltons
Bos1: Bet one suppressor1
Gos28: Golgi SNARE with a size of 28 kilodaltons
Gos1: Golgi SNAP receptor complex member 1
Vti1: Vesicular transport through interaction with t-SNARE
NPSN: Novel Plant SNARE
Use1: Unconventional SNARE in the ER1
Sft1: Suppressor of Sed5 t-SNARE
Bet1: Blocked Early in Transport
Gs15: Golgi SNARE with a size of 15 kilodaltons
Tlg1/2: t -SNARE affecting a late Golgi compartment protein
Nyv1: New Yeast v-SNARE
